# Functional remodeling of intraperitoneal macrophages by oncolytic adenovirus restores anti-tumor immunity for peritoneal metastasis of gastric cancer

**DOI:** 10.1016/j.omton.2024.200806

**Published:** 2024-04-24

**Authors:** Motoyasu Tabuchi, Satoru Kikuchi, Hiroshi Tazawa, Tomohiro Okura, Toshihiro Ogawa, Ema Mitsui, Yuta Une, Shinji Kuroda, Hiroki Sato, Kazuhiro Noma, Shunsuke Kagawa, Toshiaki Ohara, Junko Ohtsuka, Rieko Ohki, Yasuo Urata, Toshiyoshi Fujiwara

**Affiliations:** 1 Department of Gastroenterological Surgery, Okayama University Graduate School of Medicine, Dentistry and Pharmaceutical Sciences, Okayama 700-8558, Japan; 2 Center for Innovative Clinical Medicine, Okayama University Hospital, Okayama 700-8558, Japan; 3 Department of Pathology and Experimental Medicine, Okayama University Graduate School of Medicine, Dentistry and Pharmaceutical Sciences, Okayama 700-8558, Japan; 4 Laboratory of Fundamental Oncology, National Cancer Center Research Institute, Tokyo 104-0045, Japan; 5 Oncolys BioPharma, Inc., Tokyo 106-0032, Japan

**Keywords:** MT: Regular Issue, peritoneal metastasis, adenovirus, gastric cancer, macrophage, p53, immune checkpoint inhibitor

## Abstract

Intraperitoneal tumor-associated macrophages (TAMs) are involved in evading anti-tumor immunity and promoting the peritoneal metastasis (PM) of gastric cancer (GC). Oncolytic viruses are known to induce the activation of host anti-tumor immunity in addition to tumor lysis. This study investigated whether a wild-type *p53*-loading telomerase-specific oncolytic adenovirus (OBP-702) could elicit the remodeling of intraperitoneal macrophages and enhance the efficacy of immune therapy. Increased numbers of CD163 TAMs and few CD8^+^ lymphocytes were immunohistochemically observed in clinical samples with PM, which suggested that TAMs were associated with the suppression of anti-tumor immunity. OBP-702 induced immunogenic cell death and upregulated PD-L1 expression in human and murine GC cell lines. Intraperitoneal administration of OBP-702 increased recruitment of CD8^+^ lymphocytes into the PM via the functional remodeling of intraperitoneal macrophages from TAM toward a pro-inflammatory phenotype, resulting in significantly suppressed tumor growth for the *in vivo* model. Furthermore, the combination of intraperitoneal OBP-702 with anti-programmed cell death-1 antibody enhanced anti-tumor immunity and prolonged the survival of mice bearing PM. Intraperitoneal immunotherapy using OBP-702 restores anti-tumor immunity via the remodeling of intraperitoneal macrophages in addition to direct tumor lysis and cooperates with immune checkpoint inhibitors to suppress PM in GC.

## Graphical abstract

**Figure:**
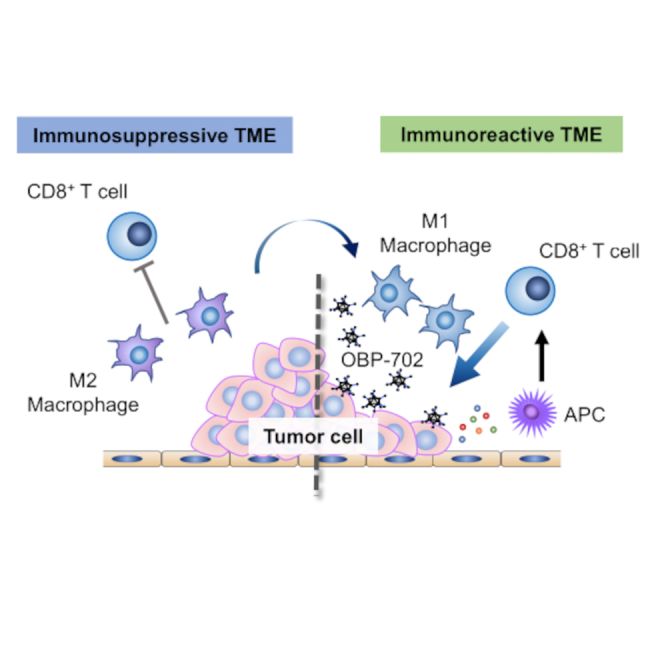

## Teaser

Kikuchi and colleagues increased intraperitoneal TAMs are associated with the suppression of anti-tumor immunity in PM. Intraperitoneal immunotherapy using p53-armed oncolytic Ad (OBP-702) increased the recruitment of tumor-infiltrating lymphocytes (TILs) via the functional remodeling of intraperitoneal macrophages from TAMs toward a pro-inflammatory phenotype and enhanced the efficacy of ICIs.

## Introduction

Peritoneal metastasis (PM) among of the most frequent forms of distant metastasis and recurrence of advance gastric cancer (GC). As such, PM is considered an extremely poor prognostic factor and curative treatments remain lacking, despite recent advances in anti-tumor modalities such as immunotherapy.^1^^,^^2^^,^^3^^,^^4^ The peritoneal cavity represents an immune environment separated from systemic immunity. In this environment, abundant resident macrophages, B cells, predominantly CD8^+^ over CD4 T cells and relatively abundant dendritic cells (DCs), as well as abundant soluble factors, orchestrate a robust immune environment. However, PM drastically changes the phenotypes of lymphocytes and macrophages in the peritoneal cavity, accompanied by changes to the extracellular matrix and fibroblasts in the tumor microenvironment (TME), which enhances the development and progression of intraperitoneal tumors.^5^^,^^6^ Peritoneally disseminated tumor cells inactivate DCs and elicit tumor-associated macrophages (TAMs) and T cell exhaustion in the peritoneal cavity.^7^^,^^8^ We have previously shown that intraperitoneal CD163^+^ TAMs were significantly more frequent in GC patients with PM compared with those without, associated with the development and progression of PM via interleukin (IL)-6 secretion.^9^ Programmed cell death ligand 1 (PD-L1) overexpression on the surface of tumor cells allows evasion of attacks by cytotoxic CD8^+^ T cells.^10^ The unique TME of the peritoneal cavity impedes the efficacy of conventional systemic chemotherapeutic agents. Moreover, the peritoneal-plasma barrier means that intravenously administered chemotherapeutic agents have difficulty penetrating the peritoneal cavity.^11^ Intraperitoneal administration of chemotherapeutic agents has, therefore, been performed for patients with PM in several phase 3 trials, showing superior survival benefits compared with systemic chemotherapy because the peritoneum, the predominant site of the tumor in ovarian cancer as well as GC, could receive continuous exposure to high concentration of chemotherapeutic agents while other organs, including bone marrow, were relatively spared in intraperitoneal treatment.^12^^,^^13^^,^^14^

Cancer immunotherapies such as immune checkpoint inhibitors (ICIs) have recently been developed and are becoming established as promising and effective treatment strategies for several advanced malignant tumors, including GC.^15^^,^^16^ However, ICIs have shown clinical benefits as a monotherapy for only a limited population of GCs, particularly with regard to PM.^17^^,^^18^^,^^19^ Novel immunotherapeutic agents that activate intraperitoneal tumor immunity in combination with ICIs are needed to overcome this limitation.

Oncolytic virotherapy (OV) is considered a novel type of immunotherapy for cancers.^20^^,^^21^^,^^22^ Selective tumor cell lysis by OV induces immunogenic cell death (ICD) with the release of tumor-associated antigens in addition to direct tumor killing. The released tumor-associated antigens are presented by DCs and other antigen-presenting cells and induce antigen-specific T cell responses. Moreover, OV induces inflammation and stimulates the secretion of cytokines and chemokines that activate immune cells into the TME treated by OV. These composite immune responses induce innate and adaptive anti-tumor immunity.^20^^,^^21^^,^^22^^,^^23^^,^^24^ As a result, OV is also recognized as an ideal means of enhancing anti-tumor immunity in combination with ICIs.^20^^,^^25^

OBP-301 (suratadenoturev) is a telomerase-specific replication-competent oncolytic adenovirus (Ad) that drives the E1A and E1B genes for viral replication under control of the human telomerase reverse transcriptase (hTERT) promoter, which induces tumor-specific lysis in several human cancer cells.^26^ In a phase 1 study, the safety and biological activity of the intra-tumoral administration of OBP-301 was confirmed in patients with several types of solid tumors in the United States.^27^ The safety and clinical efficacy of OBP-301 in combination with ionizing radiation was also confirmed in a phase 1 and 2 clinical trial for patients with esophageal cancer in Japan.^28^ As a tumor suppressor gene, *p53* is frequently inactivated in several human cancers, including GC,^29^ so *p53*-introducing gene therapy has been considered a potentially effective treatment strategy for cancers. Ad-mediated *p53* gene therapy has been performed in patients with a variety of cancers and its feasibility has been confirmed.^30^^,^^31^ We have further developed OBP-702 as a *p53*-expressing oncolytic Ad in which the wild-type *p53* gene expression cassette was inserted into the E3 region of OBP-301.^32^ We have recently shown that OBP-702 enhanced the anti-tumor efficacy of anti-programmed cell death 1 antibody (anti-PD-1 Ab) in a syngeneic mouse model of pancreatic cancer via the enhancement of ICD and CD8^+^ T cell infiltration into tumors.^33^

In the present study, greater expression of CD163^+^ TAMs were confirmed in the immunohistochemical analysis of clinical samples of PM, which might be associated with progression of PM and impede the efficacy of conventional therapy. We investigated whether OBP-702 could induce ICD in human and murine GC cells *in vitro* and whether intraperitoneal administration of OBP-702 could change the intraperitoneal TME to an immune-reactive state in an *in vivo* xenograft PM mouse model. Furthermore, intraperitoneal administration of OBP-702 enhanced anti-PD-1 Ab to suppress PM via the enhancement of anti-tumor immunity.

## Results

### CD163^+^ TAMs contribute to the development of PM and immune suppression in intraperitoneal TME

**Figure 1:**
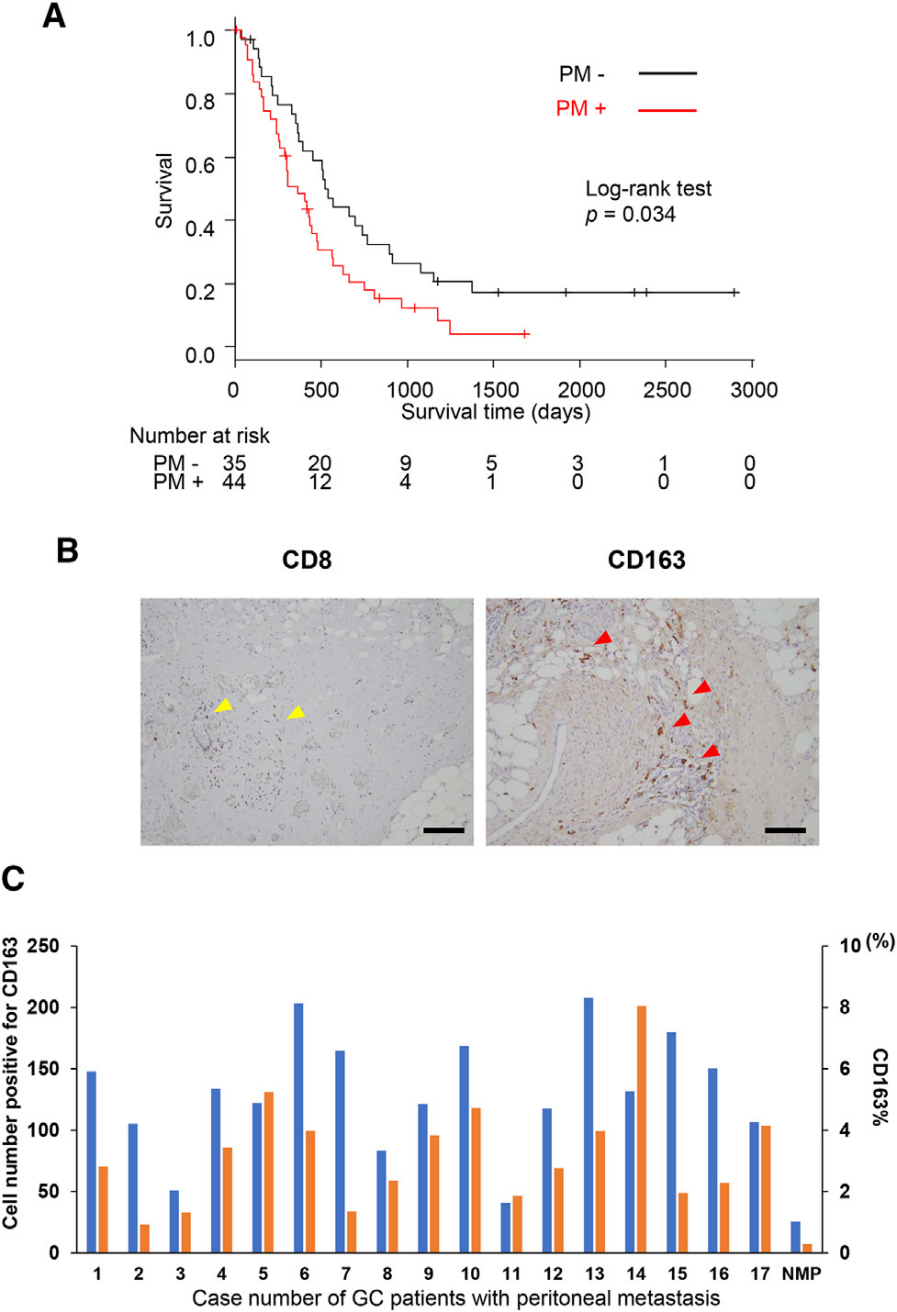
Kaplan-Meier survival curve for consecutive 79 GC patients with stage IV according to the presence of PM The median OS in the patients with PM was 364 days, which was significantly shorter than these without PM (*p* = 0.034)(A). Analysis of CD8^+^ T cells and CD163^+^ macrophages infiltrating the peritoneal tumor in clinical samples of PM by immunohistochemistry. (B) Representative microscopic images with anti-CD8 and anti-CD163 staining. Yellow arrowhead, CD8^+^ cells. Red arrowhead, CD163+ cells. Scale bars, 200 μm. (C) Enumeration of CD163^+^ macrophages in PMs resected from 17 GC patients and non-cancer parts of peritoneal tissue. The blue bars show CD163^+^ cells for each case and the orange bars show the percentage area of CD163-positive cells (CD163%) for each case.

The prognosis of GC patients with PM is extremely poor. We compared the overall survival (OS) rates of consecutive 79 stage IV GC patients between with or without PM. Forty-four stage IV GC patients with PM and 35 stage IV GC patients without PM were included in the analysis. The median OS in the patients with PM was 364 days, which was significantly shorter compared with the OS of these without PM (*p* = 0.034) (Figure 1A).

To investigate the TME in the PM of GC, we performed immunohistochemical analysis of surgically resected peritoneal disseminated nodules from 17 GC patients. In all samples, increased CD163^+^ macrophages (TAMs) were observed around cancer cells, whereas few CD8^+^ T cells were observed (Figures 1B and 1C). The number of infiltrating CD163^+^ macrophages into tumor was counted under microscopy. Counts and CD163% in each case were calculated at four sites for each tissue, then the average was calculated. Similarly, the non-cancer part of the peritoneal tissue was investigated as a basal count of CD163^+^ macrophages. The mean number and positive area of CD163^+^ macrophages from three non-cancer parts of peritoneal tissue were 25.7 ± 20.8 and 0.294% ± 0.115%. The number and the area of CD163^+^ macrophages in all 17 patients with PM were higher than non-cancer part (Figure 1C). These results suggest that CD163^+^ TAMs are related to the development and progression of PM and immune suppression in the intraperitoneal TME.

### Cytotoxic effect of OBP-702 against murine and human GC cells

**Figure 2:**
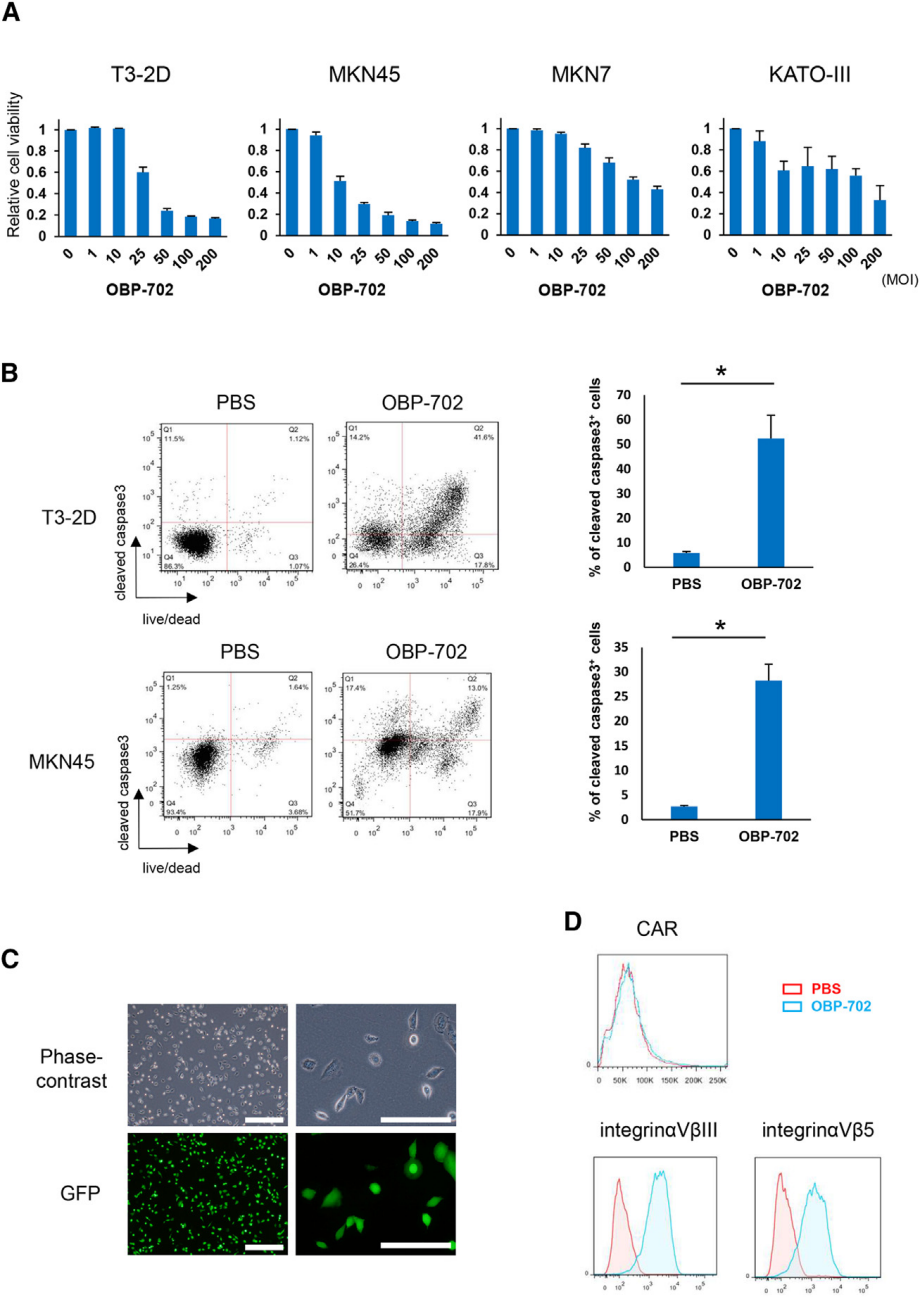
Cytotoxic effect of OBP-702 against murine and human GC cells (A) Murine GC cells, T3-2D and human GC cells, MKN45, MKN7, and Kato-III were infected with OBP-702 at the indicated MOIs for 3 days. Cell viability was quantified using the XTT assay. Cell viability in a mock-treated group was considered 1.0 and relative cell viability was calculated. Data are expressed as mean ± SD (*n* = 5). (B) Flow cytometry analysis for cleaved caspase-3. T3-2D cells were infected with OBP-702 at 50 MOI for 72 h and stained with Zombi NHR and cleaved caspase-3. Dead cells after treatment with OBP-702 showed significantly increased expression of cleaved caspase-3. (C) T3-2D cells were infected with OBP-401 at 10 MOI for 24 h. GFP expression of infected cells was assessed by fluorescence microscopy. Scale bar, 500 μm (left), 200 μm (right). (D) Flow cytometry analysis for CAR or integrin expression on the surface of T3-2D cells at 48 h after 50 MOI of OBP-702 infection.

To evaluate the antitumor effects of OBP-702 against human and murine GC cells, MKN45, MKN7, KATO-III, and T3-2D cells were treated with OBP-702. Cytotoxic effects were confirmed in all GC cells in a dose-dependent manner after infection with OBP-702 by the induction of apoptosis as shown by the upregulation of cleaved caspase-3 (Figures 2A and 2B). OBP-401 is an Ad variant of OBP-301 that allows monitoring of viral replication in cancer cells via GFP expression. Murine T3-2D cancer cells were treated with OBP-401 to confirm the infection and replication ability of oncolytic Ad in T3-2D. Higher GFP expression was confirmed in T3-2D cells 24 h after OBP-401 infection (Figure 2C). Ad uptake into cells was mainly established by endocytosis and micropinocytosis through coxsackievirus and Ad receptor (CAR) or integrin αVβ5 on the cell surface. Although CAR expression was not observed, higher integrin αVβ5 expression was observed on the T3-2D surface (Figure 2D). These results suggest that OBP-702 infected T3-2D by endocytosis and micropinocytosis through integrin αVβ5.

### Active release of immunogenic molecules and upregulation of PD-L1 expression by OBP-702 treatment in GC cells

**Figure 3:**
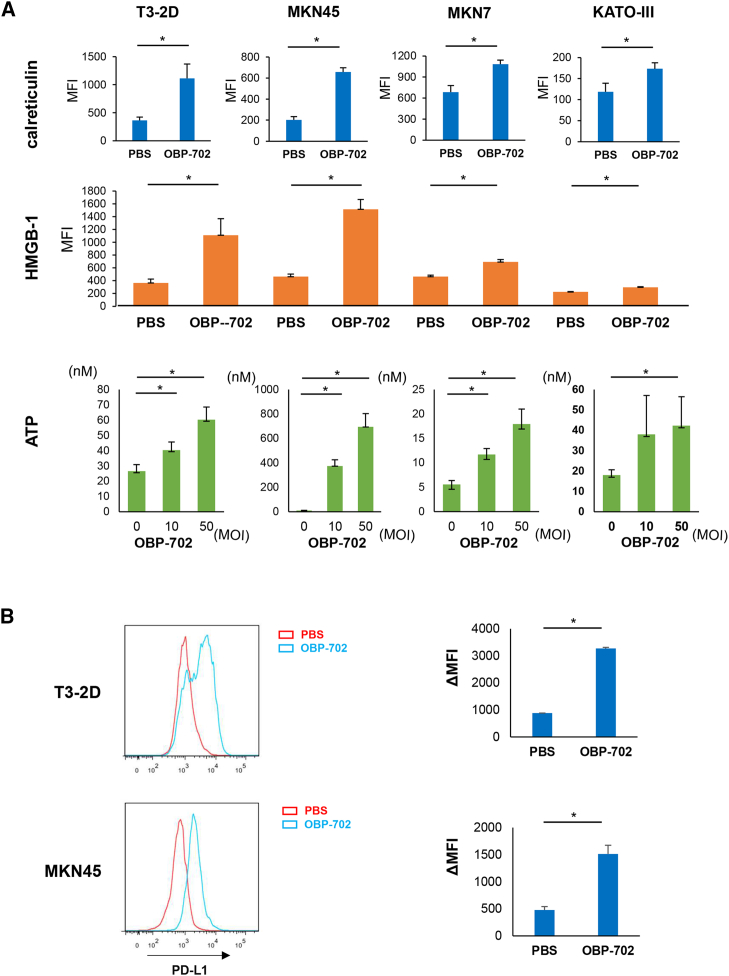
Active release of immunogenic molecules and upregulation of PD-L1 expression by OBP-702 treatment in GC cells (A) Expressions of cell surface calreticulin and intracellular HMGB-1 expression in several GC cells were measured using flow cytometry analysis 24 h after 50 MOI of OBP-702 treatment. Extracellular ATP secreted from several GC cells was measured using a luminescence assay 24 h after OBP-702 treatment (0, 10, and 50 MOI). (B) Flow cytometric analysis for PD-L1 expression on the surface of T3-2D and MKN45 cells 72 h after 50 MOI of OBP-702 infection.

Oncolytic virus treatment has been reported to induce ICD in cancer cells because cancer cells infected with oncolytic virus release both pathogen-associated molecules and damage-associated molecules, such as ATP, calreticulin, and high mobility group box 1 (HMGB-1). OBP-702 infection induced an increase in ATP release, upregulation of calreticulin expression on the surface of GC cells, and intracellular HMGB-1 expression (Figure 3A). Interestingly, OBP-702 infection also upregulated PD-L1 expression on the surface of GC cells; the higher PD-L1 expression in the tumors has been generally reported to show good reactivity to ICIs^34^^,^^35^ (Figure 3B). Furthermore, malignant ascites was collected from a stage IV GC patient with PM and incubated with OBP-702 at a multiplicity of infection (MOI) of 50 for 96 h. PD-L1 expression on the surface of EpCAM^+^ cells and dead cells with EpCAM^+^ were increased after OBP-702 infection, similar to the *in vitro* experiment (Figure S1A).

### Intraperitoneal OBP-702 treatment restored intraperitoneal anti-tumor immunity in the PM model

**Figure 4:**
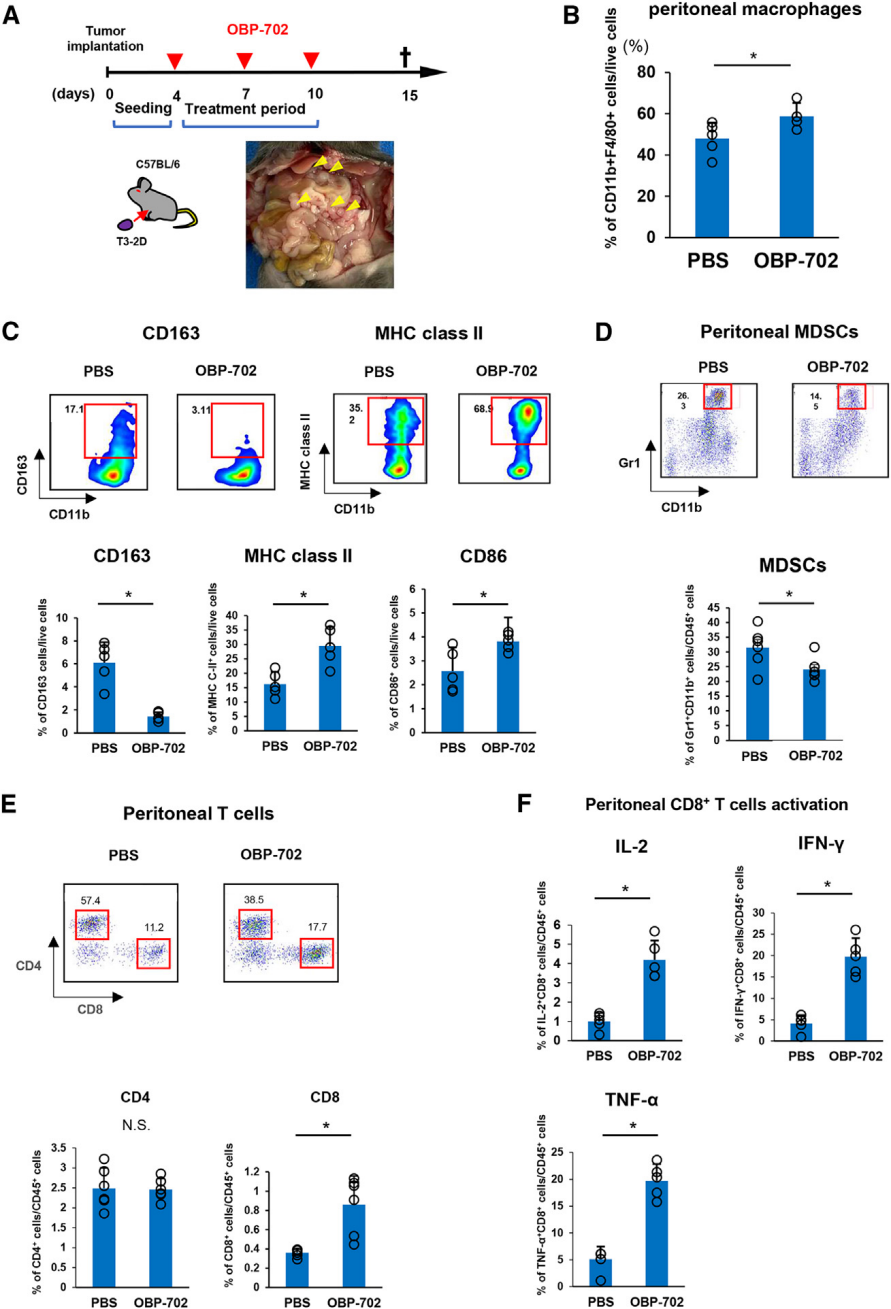
Intraperitoneal OBP-702 treatment restores intraperitoneal anti-tumor immunity T3-2D cells were intraperitoneally inoculated into C57BL/6 mice treated with intraperitoneal administration of PBS or OBP-702 (1 × 10^8^ PFU/body) three times. (A) Schematic of the treatment schedule. Red arrowheads show the timing of treatment with OBP-702 and the black cross indicates sacrifice. Representative image of peritoneally disseminated tumor nodules (yellow arrowhead). (B) Flow cytometric analysis of macrophages in intraperitoneal lavage after PBS or OBP-702 treatment. Macrophages were defined as CD11b^+^F4/80^+^ cells. OBP-702 treatment significantly increased the total number of intraperitoneal macrophages. (C) OBP-702 treatment altered the proportion of intraperitoneal macrophages; macrophages expressing MHC C-II and CD86 were significantly increased, while those expressing CD163 were significantly decreased. (D and E) Flow cytometric analysis of MDSCs, CD4^+^ T cells, and CD8^+^ T cells in intraperitoneal lavage after PBS or OBP-702 treatment. MDSCs were defined as CD45^+^/CD11b^+^/Gr1^+^ cells and T cells as CD45^+^/CD3^+^ cells. OBP-702 treatment significantly decreased the proportion of intraperitoneal MDSCs and increased CD8^+^ T cells. (F) Flow cytometric analysis for the activation of CD8^+^ T cells in intraperitoneal lavage after PBS or OBP-702 treatment. OBP-702 treatment significantly increased the proportions of IL-2^+^, IFN-γ^+^, and TNF-α^+^ T cells.

To investigate how intraperitoneal administration of OBP-702 affects the intraperitoneal TME, we performed intraperitoneal administration of OBP-702 into the orthotopic PM mouse model using T3-2D. A total of three intraperitoneal administrations of PBS or OBP-702 were performed and peritoneal lavage fluid was collected 15 days after tumor inoculation. Intraperitoneal immune cells were then analyzed by flow cytometry (Figure 4A).

Regarding innate immunity, total macrophage counts were significantly increased after intraperitoneal OBP-702 treatment compared with PBS administration (Figure 4B). OBP-702 treatment increased the proportion of macrophages expressing major histocompatibility complex class II and CD86 as representative markers of M1-like anti-tumoral macrophages and decreased the proportion of CD163^+^ M2-like pro-tumoral macrophages (Figure 4C). Further, malignant ascites was collected from a stage IV GC patient with PM and incubated with OBP-702 at 50 MOI for 96 h. OBP-702 increased the proportion of macrophages expressing CD86 and decreased the proportion expressing CD163, similar to results in the mouse model (Figure S1B). Next, we evaluated how OBP-702 treatment could change the number of myeloid-derived suppressor cells (MDSCs) that were reported to be associated with the suppression of anti-tumor immunity.^36^ Even though few MDSCs were present in the peritoneal cavities of mice without PM (Figure S2), intraperitoneal MDSCs were markedly increased in mice with PM. Intraperitoneal OBP-702 treatment significantly decreased intraperitoneal MDSCs (Figure 4D).

Regarding adaptive immunity in the peritoneal cavity, intraperitoneal OBP-702 treatment significantly increased the proportion of CD8^+^ cytotoxic T cells, although the proportion of CD4^+^ T cells was similar to that with PBS administration (Figure 4E). Furthermore, intraperitoneal OBP-702 treatment significantly increased the numbers of IL-2-, interferon (IFN)-γ-, and tumor necrosis factor (TNF)-α-secreting cytotoxic CD8^+^ T cells (Figure 4F). These results indicate that intraperitoneal OBP-702 treatment improved the immunosuppressive environment by PM through enhanced innate and adaptive immunity.

### Intraperitoneal OBP-702 treatment increases inhibitory checkpoint molecules in immune cells

**Figure 5:**
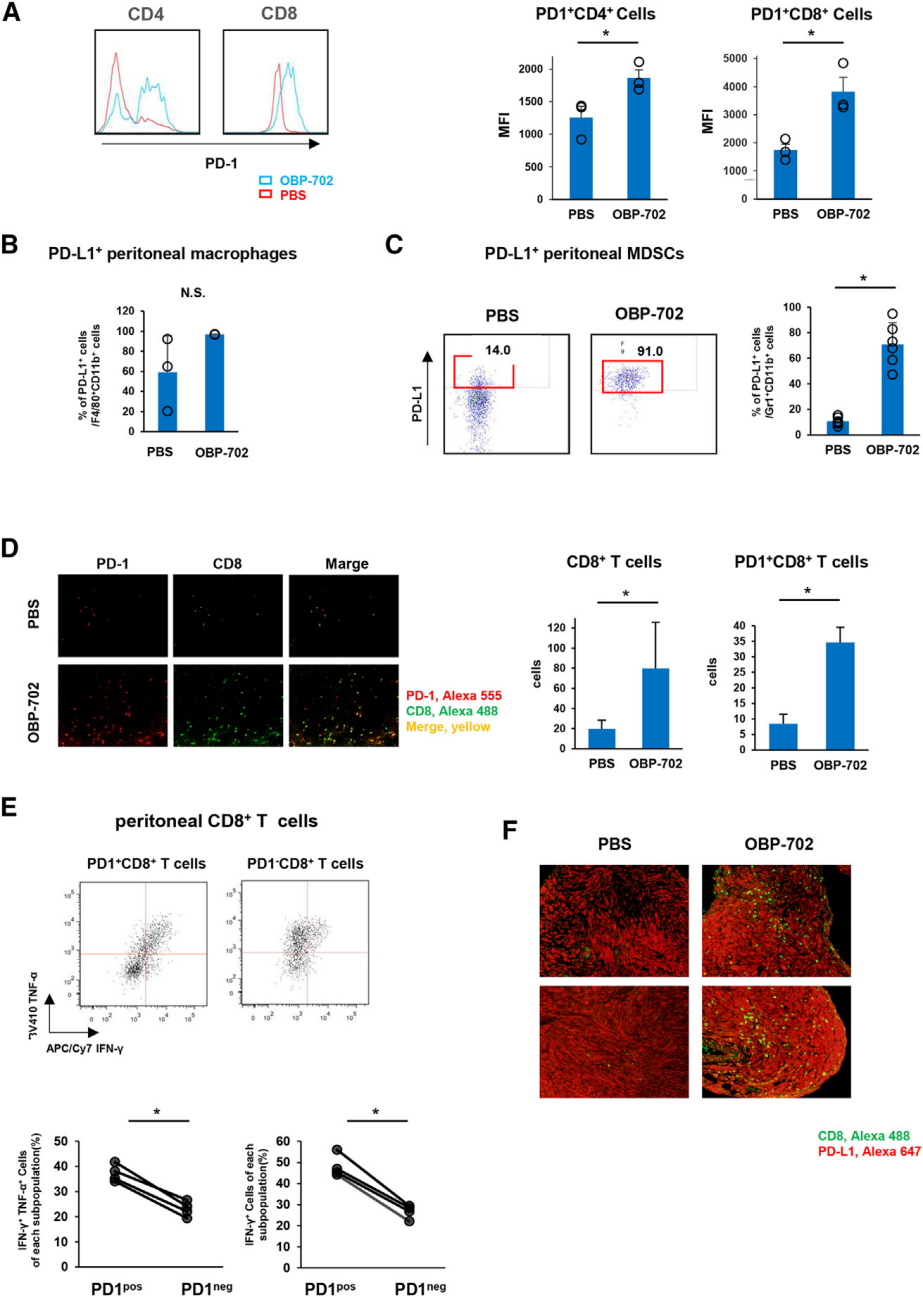
Intraperitoneal OBP-702 treatment increases inhibitory checkpoint molecules in immune cells (A) Intraperitoneal lavage fluid was collected after treatment with PBS or OBP-702 and subjected to flow cytometric analysis for PD-1 expression on CD4^+^ and CD8^+^ T cells. (B and C) PD-L1 expression on intraperitoneal macrophages and MDSCs after PBS or OBP-702 treatment. (D) Representative images of CD8 (green) and PD-1 (red) staining in intraperitoneal T3-2D tumor tissues after PBS or OBP-702 treatment (left). Mean numbers of CD8^+^ TILs and PD-1^+^ CD8^+^ TILs were significantly increased after OBP-702 treatment (right). (E) Representative flow cytometry plots and summary data show the frequencies of both of IFN-γ- and TNF-α-producing cells and IFN-γ-producing cells in PD-1^+^ and PD-1− subpopulations of intraperitoneal CD8^+^ T cells after PBS or OBP-702 treatment. (F) Representative images of CD8 (green) and PD-L1 (red) staining in intraperitoneal T3-2D tumor tissues after PBS or OBP-702 treatment.

To further evaluate the impact of intraperitoneal OBP-702 treatment on intraperitoneal anti-tumor immunity, we analyzed the expression of inhibitory checkpoint molecules such as PD-1 and PD-L1 on immune cells. Intraperitoneal OBP-702 treatment significantly increased PD-1 expression on intraperitoneal CD4^+^ and CD8^+^ T cells and PD-L1 expression on intraperitoneal macrophages and MDSCs (Figures 5A–5C). Although the number of tumor-infiltrating CD8^+^ T cells was increased after OBP-702 treatment, most of these CD8^+^ T cells expressed PD-1 (Figure 5D).

Next, we evaluated whether PD-1-expressing CD8^+^ T cells had effector potential or were exhausted cells. Secretions of IFN-γ and TNF-α were significantly increased in PD-1-expressing CD8^+^ T cells compared with CD8^+^ T cells without PD-1 expression (Figure 5E). The number of CD8^+^ T cells infiltrating into peritoneal tumor was increased after OBP-702 treatment (Figure 5F). These results indicate that PD-1-expressing CD8^+^ T cells after OBP-702 treatment have effector potential to attack tumor cells.

Although intraperitoneal OBP-702 treatment increased the expression of inhibitory checkpoint molecules on immune cells, these results might reflect a restoration of effector functions in CD8^+^ T cells and combination with ICIs such as anti-PD-1 Ab might enhance the efficacy of OBP-702 against the PM.

### Intraperitoneal OBP-702 treatment combined with anti-PD-1 Ab eradicates PM of GC via activation of anti-tumor immunity

**Figure 6:**
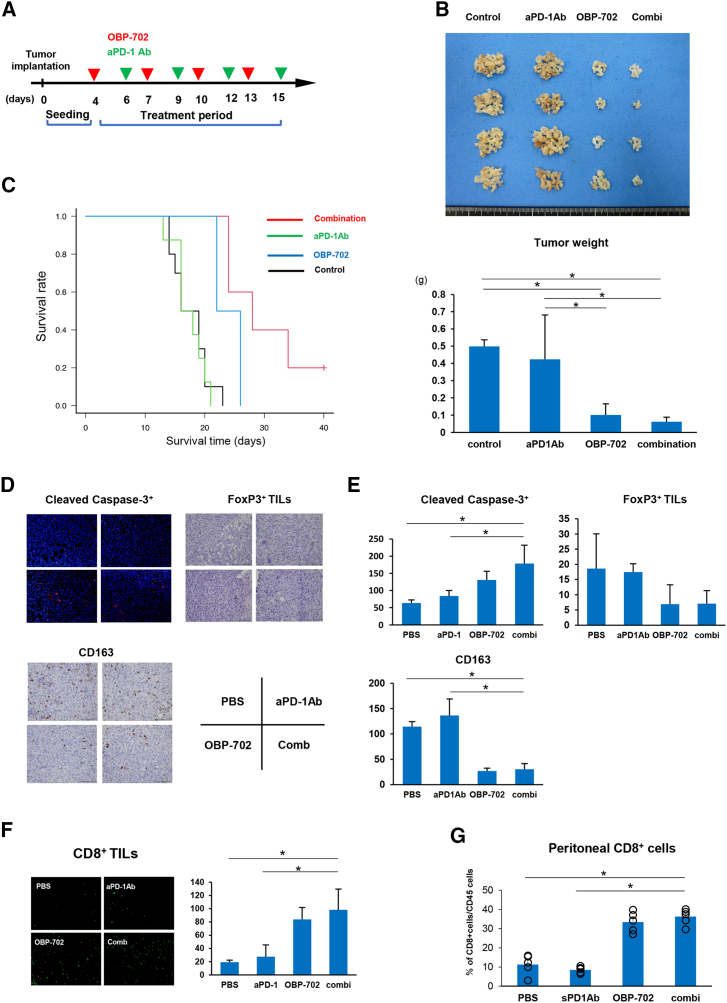
Intraperitoneal OBP-702 treatment combined with anti-PD-1 Ab eradicates PM of GC via activation of anti-tumor immunity (A) Schematic for the treatment schedule. Orthotopic mouse model of PM treated by intraperitoneal administration of PBS, anti-PD-1 Ab, OBP-702 or the combination of OBP-702 and anti-PD-1 Ab four times. Red arrowheads show the timing of treatments with OBP-702 and green arrowheads show treatments with anti-PD-1 Ab. (B) Macroscopic images of peritoneal nodules treated with PBS, anti-PD-1 Ab, OBP-702, or the combination of OBP-702 and anti-PD-1 Ab and the total weight of peritoneal nodules in each group (*n* = 4). (C) Kaplan-Meier curves for OS in mice treated with each treatment. (D) Representative images of immunohistochemical staining for cleaved caspase-3, Foxp3, and CD163 in T3-2D tumor tissues treated with each treatment. (E) The mean numbers of cells expressing cleaved caspase-3, Foxp3, and CD163 in peritoneal tumors treated with each treatment. The mean cell numbers were calculated from three selected fields in each mouse, from a total of five mice per group. (F) Representative images of immunohistochemical staining for CD8^+^ TILs in T3-2D tumor tissues treated with each treatment. The mean number of CD8^+^ TILs was calculated from three selected fields in each mouse, from a total of five mice per group. (G) Intraperitoneal CD8^+^ T cells were analyzed by flow cytometry of peritoneal lavage exposed to each treatment.

Although we showed that intraperitoneal OBP-702 treatment restored anti-tumor immunity, PD-1 expression on CD8^+^ T cells was also upregulated in orthotopic PM models. We, therefore, combined anti-PD-1 Ab with OBP-702 to confirm whether anti-PD-1 Ab could enhance the efficacy of intraperitoneal OBP-702 treatment. Either intraperitoneal OBP-702 and/or anti-PD-1 Ab treatment was performed for mice bearing PM, as indicated in the study protocol (Figure 6A). Anti-PD-1 Ab monotherapy suppressed the total volume of PM by 15%, compared with 79.6% with OBP-702 monotherapy and 87.5% with combination therapy (Figures 6B and S3). The combination of intraperitoneal OBP-702 treatment and anti-PD-1 Ab prolonged the OS of mice bearing PM compared with those in other groups (Figure 6C). Moreover, immunohistochemical analysis of PM after treatment showed that combination therapy significantly increased the number of cleaved caspase-3-positive cells compared with monotherapy, indicating that combination therapy has better anti-tumor effects against PM. The analysis of intraperitoneal TME after treatment showed that intraperitoneal OBP-702 treatment and combination therapy significantly decreased Foxp3^+^ TILs and CD163 cells in PM compared with controls (Figures 6D and 6E). Combination therapy also increased the number of CD8^+^ TILs and intraperitoneal CD8^+^ T cells compared with controls and anti-PD-1 monotherapy (Figures 6F and 6G). These results indicate that intraperitoneal OBP-702 treatment in combination with anti-PD-1 Ab could further improve intraperitoneal immune environment against PM and shows promising therapeutic potential against PM.

### Safety and feasibility of intraperitoneal OBP-702 treatment

**Figure 7:**
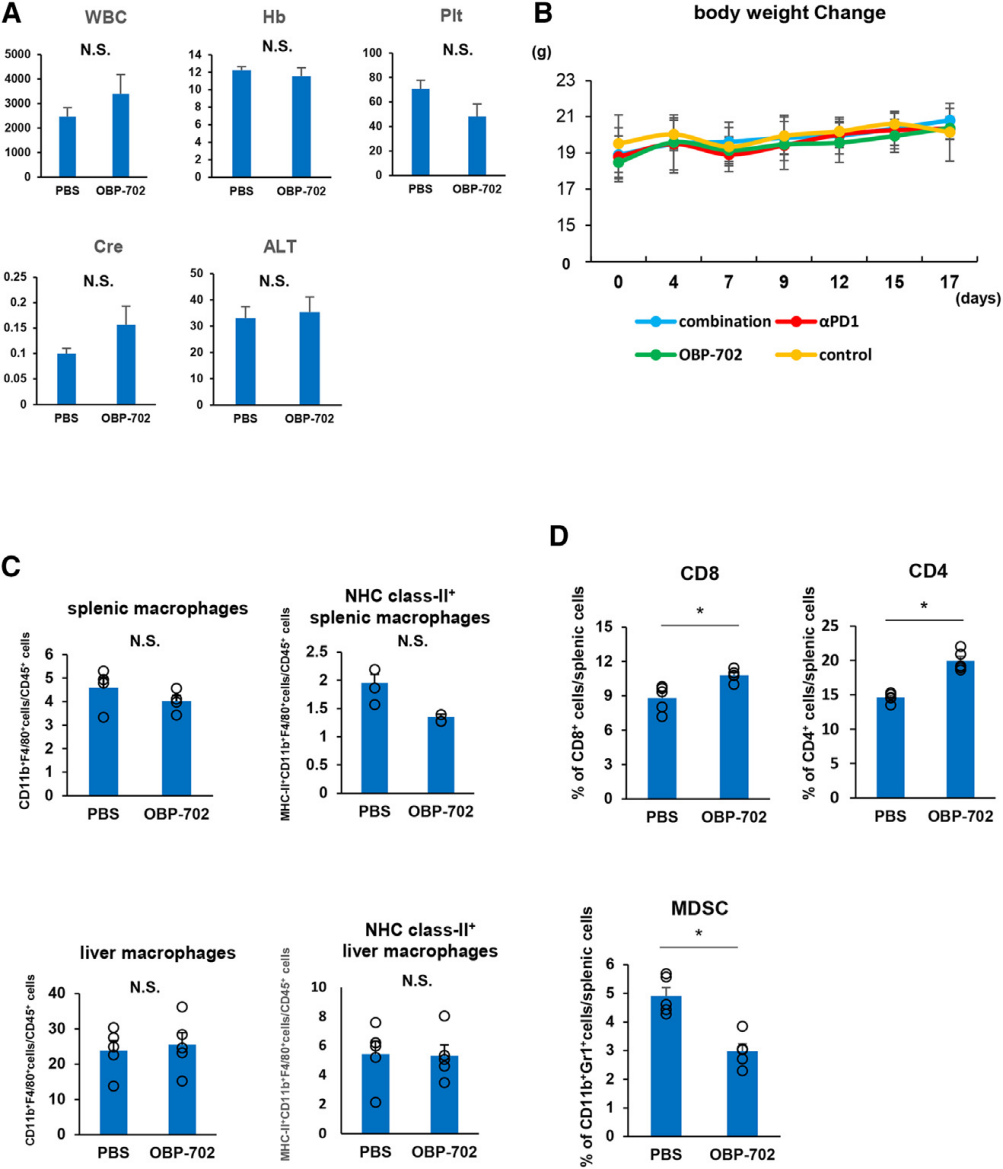
Safety and feasibility of intraperitoneal OBP-702 treatment (A) Biochemical data for blood from mice 3 days after intraperitoneal administration of PBS or OBP-702. (B) Body weight changes in mice treated with each treatment. (C) Total numbers of all macrophages and MHC class II^+^ macrophages in the spleen and liver of mice treated with PBS or OBP-702. (D) Flow cytometric analysis of MDSCs and CD8^+^ and CD4^+^ T cells in the spleen after PBS or OBP-702 treatment.

Finally, we investigated the safety and systemic influence of intraperitoneal OBP-702 treatment. Mouse blood samples were collected after two cycles of intraperitoneal OBP-702 treatment and compared laboratory data with those from untreated mice. No significant differences were seen regarding white blood cell count, hemoglobin level, platelet count, serum creatinine, or aminotransferases (Figure 7A). Body weight changes were similar among groups during treatment (Figure 7B). Furthermore, we confirmed the influence of intraperitoneal OBP-702 treatment on the liver and spleen. No significant increase in macrophages in the liver and spleen was observed in the intraperitoneal OBP-702 treatment; in particular, MHC class II and inflammatory macrophages were not increased (Figure 7C). However, CD8^+^ and CD4^+^ T cells were significantly increased and MDSCs were significantly decreased in the spleen after intraperitoneal administration of OBP-702 (Figure 7D).

## Discussion

PM is a common form of distant metastasis for GC, but is incurable under current systemic treatment regimens because conventional chemotherapeutic agents administered systemically cannot effectively reach the intraperitoneal cavity. Intraperitoneal therapy, including intraperitoneal chemotherapy in combination with systemic chemotherapy and hyperthermia, represents a promising approach to PM.^12^^,^^13^^,^^14^^,^^37^ OV is a promising candidate as intraperitoneal therapy for PM, as well as intraperitoneal chemotherapy. The safety and feasibility of intraperitoneal administration of OVs in patients with advanced ovarian cancer have been confirmed in some phase 1 clinical trials.^38^^,^^39^ We recently revealed that the intraperitoneal administration of OBP-702 had therapeutic effects against cancer-associated fibroblasts and synergistically suppressed PM in a xenograft mouse model in combination with paclitaxel.^40^ The current study showed that intraperitoneal administration of OBP-702 restored intraperitoneal anti-tumor immunity for PM and synergistically suppressed PM in combination with anti-PD-1 Ab.

Although we have previously shown that CD163^+^ macrophages were significantly higher in lavage fluid from the intraperitoneal cavity of GC patients with PM compared with that from patients without PM, CD163^+^ macrophages were increased and infiltrating CD8^+^ T cells were decreased in peritoneal tumor nodules in the current study. Moreover, CD163^+^ macrophages and MDSCs were increased in the peritoneal cavity of mice bearing PM, indicating that the TME in PM is immunosuppressive.

We have shown the advantage of intraperitoneally administering OBP-702 against PM. OBP-702 selectively infected cancer cells and caused autophagy and apoptosis in PM because of the ability to achieve replication under the control of the hTERT promoter and the introduction of wild-type *p53* into cancer cells. The resulting selective cancer cell death via the induction of apoptosis and autophagy by OBP-702 increased the release of immunologic molecules including HMGB-1, ATP, and calreticulin, inducing immunologic cell death and resulting in OBP-702 restoring intraperitoneal anti-tumor immunity for PM, such as enhancement of CD8^+^ T cell infiltration into the tumor. We have previously reported that the OBP-301 variant increased the release of chemokines, such as CCL5/RANTES and CXCL10/IP-10, which were related to the recruitment of TILs after tumor cell infection.^41^ Moreover, we have previously reported that OBP-702, the *p53*-armed OV, significantly increased the release of immunologic molecules compared with OBP-301, the non-armed OV, via stronger p53-mediated apoptosis and autophagy in pancreatic ductal adenocarcinoma cells.^33^ Intraperitoneal administration of OBP-702 also altered the polarization of macrophages to an inflammatory phenotype, with M1 macrophages predominating and MDSCs decreased in the peritoneal cavity of mice bearing PM. This intraperitoneal immunologic alteration might be induced by the increased release of IFN-γ, which plays a critical role in the polarization of macrophages to an inflammatory phenotype in addition to the direct physiological response to viral infection; we have previously reported that OBP-301 treatment increased the release of IFN-γ.^42^

The intensity of PD-L1 expression in tumor tissue is generally considered a biomarker for the effect of immunotherapy, including anti-PD-1 Ab.^43^ Similar to several virus infections increasing PD-L1 expression on hematopoietic and non-hematopoietic cells to minimize collateral tissue damage, OBP-702 also increased PD-L1 expression on GC cells in the current study.^44^ Type I and III IFNs are induced in the early phase after viral infection as the first line of antiviral defense.^45^ In the late phase of acute viral infection, type II IFNs including IFN-γ and several other cytokines (including TNF-α and IL-10) are released by CD8^+^ T cells, strongly upregulating PD-L1 on various cell types.^44^ OBP-702 also increased the production of these IFNs in intraperitoneal CD8^+^ T cells. Intraperitoneal OBP-702 treatment combined with ICIs is, thus, a promising treatment strategy for PM, with significant treatment effects shown in the current study.

A phase 1 clinical trial of locoregionally injected OBP-301 was conducted in patients with several types of advanced solid tumors and the safety and feasibility of OBP-301 have already been confirmed.^27^ We also recently reported the benefits and feasibility of combination therapy with intratumoral OBP-301 injection and radiotherapy in patients with esophageal cancer unfit for standard treatment.^28^ In the current study, no significant adverse effects (including blood biochemical data, body weight, or macrophages in vital organs) were confirmed with intraperitoneal administration of OBP-702. In a preclinical study, we confirmed that OBP-301 administered intraperitoneally was specifically distributed to PMs in a xenograft model.^46^ Intraperitoneal OBP-702 treatment combined with ICIs is, therefore, likely to provide a feasible treatment strategy against PM of GC in the future.

In conclusion, we have demonstrated that CD163^+^ TAMs might contribute to PM progression and creation of an intraperitoneal immunosuppressive environment. Intraperitoneal immunotherapy by OBP-702 restores anti-tumor immunity in addition to direct tumor lysis and cooperates with ICIs to suppress PM from GC. Intraperitoneal immunotherapy by OBP-702 in combination with ICIs might provide an optimal treatment strategy against PM in GC.

## Materials and Methods

### Patients and immunohistochemistry in clinical samples

We retrospectively reviewed the medical records of consecutive 79 GC patients with stage IV disease at the department of gastroenterological surgery in Okayama University Hospital between 2014 and 2019. The clinical stage classification and pathological diagnosis were performed based on the Japanese Classification of Gastric Carcinoma.^47^ Furthermore, 17 patients with white nodules on the peritoneum, which were resected and histologically diagnosed as positive for cancer at the staging laparoscopy or the elective gastrectomy for GC were investigated regarding the TME by immunohistochemistry. The patients’ demographics and disease characteristics are shown in Table S1. First, the presence of tumor was confirmed using hematoxylin and eosin staining. Sectioned tissues were incubated with rabbit anti-CD163 monoclonal Ab (mAb) (ab182422; Abcam plc, Cambridge, UK), or mouse anti-CD8a mAb (GA623; Dako, Glostrup, Denmark) for immunohistochemistry. The immunostained slides were viewed at low magnification (×40), and four areas with high immune cells density in the tumor were selected in PM. Similarly, four areas with high immune cells density in the peritoneum including mesothelial cells were selected in the three non-cancer tissues. The number of immunostaining-positive cells was counted using ImageJ software at high magnification (×400) and the mean immunostaining-positive cells were calculated for each case. Furthermore, the percentage area of CD163-positive cells (CD163%) was calculated as (area of CD204-positive cells/measured area) ×100 using ImageJ software. The mean value obtained from each sectioned tissue was defined as the number of cells positive for CD163 or CD8. All evaluations were performed by an independent pathologist blinded to clinical information. Immunoreactive signals were visualized using 3,39-diaminobenzidine tetrahydrochloride solution, and nuclei were counterstained with hematoxylin. Sections were observed under light microscopy (BX50; Olympus, Tokyo, Japan).

### Cell lines

Three human GC cell lines were used in this study. MKN7 and MKN45 were purchased from the Japanese Collection of Research Bioresources Cell Bank and maintained in RPMI-1640 medium supplemented with 10% heat-inactivated fetal bovine serum (FBS) (Sigma-Aldrich, St. Louis, MO, USA). KATOIII cells were obtained from Health Science Research Resources Bank (Osaka, Japan) and maintained in a 1:1 mixture of Eagle’s Minimum Essential Medium and RPMI1640 supplemented with 10% FBS. T3-2D is a mouse GC cell line established by Dr. Ohki at the National Cancer Center Research Institute^48^ and kindly provided and maintained in DMEM supplemented with 10% FBS and 1% penicillin-streptomycin (100 U/mL). All media were supplemented with 100 U/mL penicillin and 100 μg/mL streptomycin. Cells were routinely maintained at 37°C in a humidified atmosphere with 5% CO_2_.

### Recombinant Ad and chemotherapeutic reagents

The recombinant, telomerase-specific, replication-competent Ad vector, OBP-301 (suratadenoturev) has been described and characterized elsewhere.^26^^,^^49^ OBP-401 (TelomeScan) is a telomerase-specific, replication-competent Ad variant into which the replication cassette and GFP expression under control of the cytomegalovirus promoter were inserted into the E3 region in OBP-301, allowing the monitoring of viral replication.^50^ OBP-702 is another Ad variant that inserts a human wild-type *p53* gene expression cassette under the control of the Egr-1 promoter into the E3 region of OBP-301. Viruses were purified by ultracentrifugation using CsCl step gradients. Viral titers were determined by plaque-forming assay using 293 cells, and the virus was stored at −80°C.

### ICIs

Anti-mouse PD-1 Ab (clone 4H2) was obtained from Ono Pharmaceutical (Osaka, Japan).

### Cell viability assay

Human (MKN7, MKN45, and KATOIII) and mouse (T3-2D) GC cells were seeded onto 96-well plates at a density of 1 ×10^3^ cells/well and cultured for 24 h before viral infection. After that, cells were infected with OBP-702 at MOIs of 0, 1, 10, 25, 50, or 100 plaque-forming units (PFU)/cell for 72 h. Cell viability was examined using the Cell Proliferation Kit II (Roche Molecular Biochemicals, Indianapolis, IN, USA), which is based on the sodium 3′-[1-(phenylaminocarbonyl)-3,4-tetrazolium]-bis(4-methoxy-6-nitro) benzene sulfonic acid hydrate (XTT) assay in accordance with the protocol from the manufacturer.

### ATP, HMGB-1, and calreticulin assays

Human (MKN7, MKN45, and KATOIII) and mouse (T3-2D) GC cells were infected with OBP-702 (0, 10, or 50 MOI) for 24 h (*n* = 5), and levels of extracellular ATP in supernatants were measured using an ENLITEN ATP assay (Promega, Madison, WI, USA), according to the protocols from the manufacturer. HMGB-1 and calreticulin assays were performed as follows. Cells were incubated with Fc block, followed by CD16/32 antibodies (Thermo Fisher Scientific, Waltham, MA, USA). Cells were stained with LIVE/DEAD Fixable Aqua Dead Cell Stain Kit (Thermo Fisher Scientific) to detect live cells. Flow Cytometry was performed on a FACSArray (BD Biosciences, San Jose, CA, USA), and analyzed by the FlowJo program (BD Biosciences, Franklin Lakes, NJ, USA). The antibodies used for flow cytometry were mouse anti-HMGB-1 Ab (MA5-16263, Invitrogen, Waltham, MA, USA) and mouse anti-Calreticulin Ab (ab22683, Abcam plc).

### Flow-cytometric analysis

Cells in the peritoneal cavity were collected using the following procedures. Mice were injected with 5 mL PBS into the peritoneal cavity, and the fluid was then collected after shaking the abdomen slightly. To minimize mouse discomfort, peritoneal fluid collection was performed under general anesthesia, followed by peritoneal lavage and sacrifice. The collected peritoneal fluid was stored on ice. Cells were extracted from peritoneal fluid by passage through a cell strainer, followed by centrifugation at 300×*g* for 10 min to remove red blood cells. The cell pellet was resuspended in 1 mL lysis buffer and centrifuged again at 400×*g* for 5 min. After washing twice with 2% FBS in PBS, cells were treated with Fc blocker (0.5 μL per 100 μL staining solution) and incubated at room temperature for 10 min. Cells were then incubated with the desired Ab in the Fc blocker solution for 30 min at room temperature. Cells were incubated with Fc block and stained using CD16/32 antibodies (Thermo Fisher Scientific) for 30 min on ice in the dark. Cleaved caspase-3 and Foxp3 were stained using Foxp3 Transcription Factor Staining (Thermo Fisher Scientific) or a BD Cytofix/Cytoperm Fixation/Permeabilization Kit (BD Biosciences, San Jose, CA, USA). Cells were stained with LIVE/DEAD Fixable Aqua Dead Cell Stain Kit (Thermo Fisher Scientific) to detect live cells. Flow cytometry was performed on a FACSArray (BD Biosciences, San Jose, CA, USA), and the results were analyzed using the FlowJo program (BD Biosciences, San Jose, CA, USA). The following antibodies were used for flow cytometry analysis to detect each target in the *in vitro* experiments. For the analysis of intraperitoneal cells, the following antibodies were used for flow cytometry analysis: PerCP-CD45, FITC-CD11b, APC-F4/80, PE/Cy7-MHC-2, and PE-CD163 to detect macrophages; PerCP-CD45, APC-CD3, APC-Cy7-CD4, FITC-CD8, BV-421-CD152, PE-CD279, BV510-CD69, BV421-CD25, and PE-PD-1 to detect CD8 and CD4 cells; and PerCP-CD45, FITC-CD11b, PE-Gr1, and APC-PD-L1 to detect MDSCs.

### Immunohistochemistry

For histological analyses, mouse peritoneal tumor nodules were removed and fixed in 10% neutralized formalin. All tissues were subsequently dehydrated in alcohol, embedded in paraffin, and sectioned for hematoxylin and eosin staining and immunohistochemical examinations. After deparaffinization and rehydration, antigen retrieval was performed by microwave irradiation in 10 mM citrate buffer (pH 6.0). Following quenching of endogenous tissue peroxidases, tissue sections were incubated with mouse anti-CD8a mAb (clone 4SM15; eBioscience, Phoenix, AZ, US), rabbit anti-PD-1 Ab (eBioscience), anti-rat Foxp3 Ab (clone FJK-16s; eBioscience), and mouse anti-CD4 mAb (lone 4SM95; eBioscience) to detect PD-1-expressing CD8 T cells and CD4 T cells. Immunoreactive signals were visualized with a 3,39-diaminobenzidine tetrahydrochloride solution, and the nuclei were counterstained with hematoxylin. Sections were viewed under a microscope (BX50; Olympus).

### Animal experiments

T3-2D cells (5 ×10^6^ cells) were inoculated into the peritoneal cavity of 6- to 8-week-old female C57BL/6 mice (CLEA Japan, Tokyo, Japan) as models of peritoneal dissemination of GC. Four days after cell inoculation, 500 μL solution containing OBP-702 (1 × 10^8^ PFU) or PBS was injected into the intraperitoneal cavity every 3 days for a total of three doses in the OBP-702 monotherapy experiment. In combination therapy, 2 days after OBP-702 injection, 20 mg/body anti-PD1 Ab was injected intraperitoneally at one and two cycles, and 10 mg/body anti-PD1 Ab was injected intraperitoneally at three and four cycles, a total of four doses of OBP-702 and anti-PD1 Ab were injected intraperitoneally.

Five mice were used for each group. All tumor nodules in the peritoneal cavity were resected and total weights were measured on day 15. The survival of each mouse was monitored, and the OS was calculated in the same treatment protocol. Animals were excluded from the experiments only if tumors did not form or if health concerns were reported. For all animal experiments, mice were randomly grouped and the measurements for tumor size were carried out blind for the groups.

Anti-cleaved caspase-3 mAb (Cell Signaling, Danvers, MA, US), anti-PD-1 mAb (eBioscience), anti-Foxp3 mAb (eBioscience), and anti-CD8a mAb (eBioscience) were used for the immunohistochemical analyses of peritoneal tumor nodules. Immunoreactive signals were visualized with a 3,39-diaminobenzidine tetrahydrochloride solution, and nuclei were counterstained with hematoxylin. Sections were viewed under light microscopy (BX50; Olympus).

### Statistical analysis

OS was calculated using the Kaplan-Meier method, with the log rank test used for comparisons between subgroups. Student’s *t* test was used to identify significant differences between groups. All data are expressed as mean ± SD. Values of *p* < 0.05 were considered statistically significant. Statistical analysis was performed using JMP version 11.2 (SAS Institute, Cary, NC, USA).

### Study approval

This study was conducted in accordance with the ethical standards of the Declaration of Helsinki and the ethical guidelines for medical and health research involving human subjects. Studies using clinical samples were approved and reviewed by the institutional review board of Okayama University Hospital (approval nos. KEN1507-031 and 2307-012). All animal experimental protocols were approved by the Ethics Review Committee for Animal Experiments of Okayama University. All animal experimental protocols were approved by the Ethics Review Committee for Animal Experiments of Okayama University (approval no. OKU-2020173).

## Data and code availability

All data generated or analyzed during this study are included in this article and its supplementary material files. Further enquiries can be directed to the corresponding author.

## Supplemental information

Document S1. Figures S1–S3 and Table S1

Document S2. Article plus supplemental information
