# Supplementary material for: Functional remodeling of intraperitoneal macrophages by oncolytic adenovirus restores anti-tumor immunity for peritoneal metastasis of gastric cancer
Source: Mol Ther Oncol. 2024 Apr 24;32(2):200806. doi: 10.1016/j.omton.2024.200806 (PMC11090911; doi:10.1016/j.omton.2024.200806)
Supplement: Document S1. Figures S1–S3 and Table S1 [file mmc1.pdf]

OMTON, Volume *32*

Supplemental information

Functional remodeling of intraperitoneal macrophages by oncolytic adenovirus restores anti-tumor immunity for peritoneal metastasis of gastric cancer

Motoyasu Tabuchi, Satoru Kikuchi, Hiroshi Tazawa, Tomohiro Okura, Toshihiro Ogawa, Ema Mitsui, Yuta Une, Shinji Kuroda, Hiroki Sato, Kazuhiro Noma, Shunsuke Kagawa, Toshiaki Ohara, Junko Ohtsuka, Rieko Ohki, Yasuo Urata, and Toshiyoshi Fujiwara

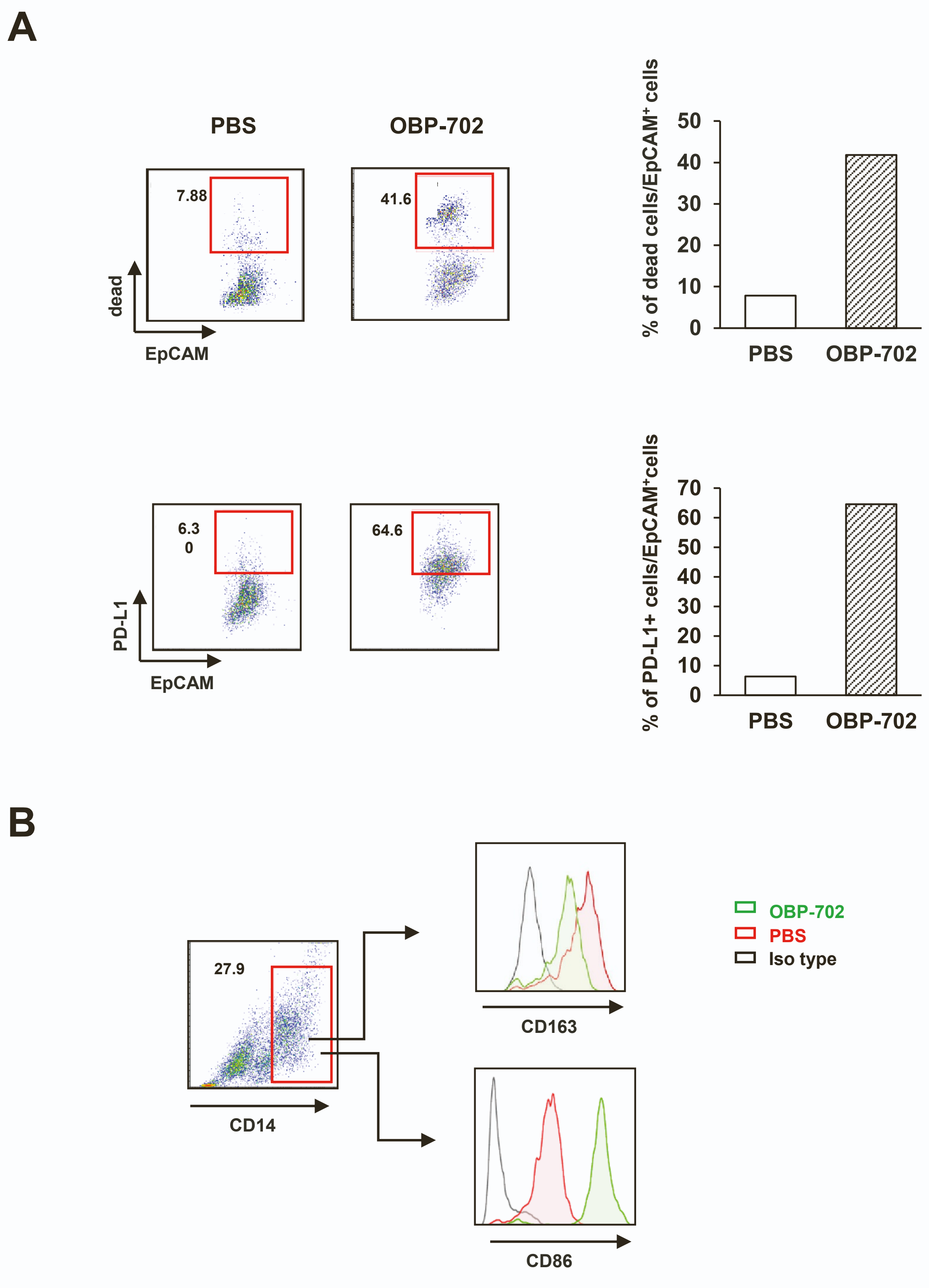

Fig. S1

Ex vivo analysis of malignant ascites of GC patients with peritoneal metastasis. Malignant ascites was collected from a Stage IV GC patients with peritoneal metastasis and incubated with OBP-702 or PBS. A) PD-L1 expression on the surface of EpCAM+ cells and dead cells with EpCAM+ were increased after OBP-702 infection. B) OBP-702 increased the proportion of macrophages expressing CD86 and decreased those expressing CD163.

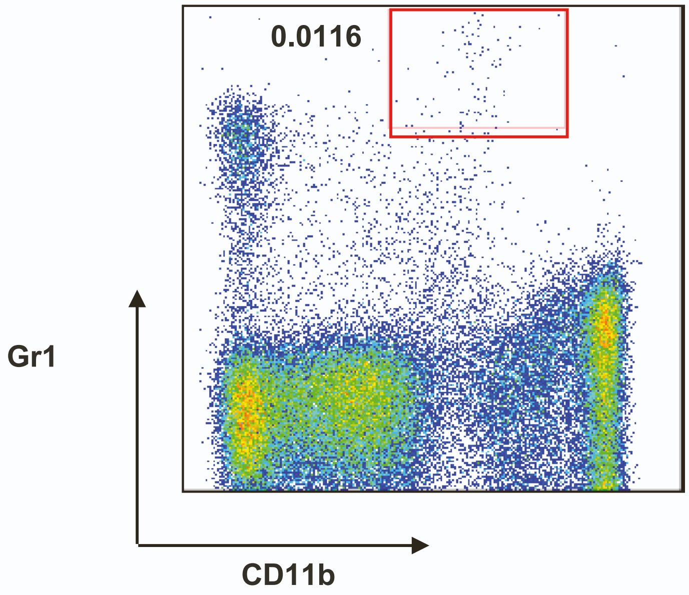

Fig. S2

Flow cytometry analysis for peritoneal Cr-1+CD11b+ cells (MDSCs) in naïve C57BL/6 mice without tumor inoculation. MDSCs were really infiltrated into the peritoneal cavity.

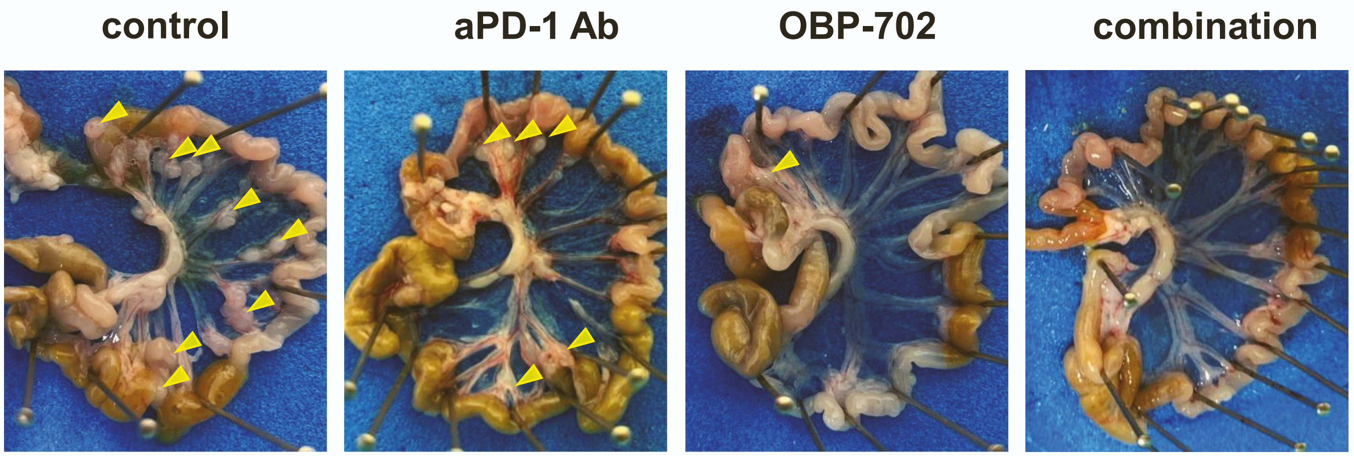

Fig. S3

Representative image of disseminated tumor nodules on the mice mesentery in each treatment. Yellow arrowheads show the tumor nodules.

Table. S1

Patients' demographics and disease characteristics of 17 GC patients with PM which was resected for diagnosis.

	number	percentage
gender		
male	8	47.1
female	9	52.9
Age		
median (range)	63 (36-81)	
Histological type		
diffuse	10	58.8
intestinal	7	41.2
Previous chemotherapy		
Yes	1	5.9
No	16	94.1
